# Cartilaginous Myringoplasty: Anatomical and Functional Results

**DOI:** 10.7759/cureus.37059

**Published:** 2023-04-03

**Authors:** Abdelouahid Taleuan, Mohamed Ridal, Hajar Elatiq, Najib Benmansour, Abdellatif Oudidi, Mohamed Amine Elalami

**Affiliations:** 1 Otolaryngology, Head and Neck Surgery, University Hospital Hassan II, FES, MAR; 2 Otolaryngology, CHU Hasssan II FES - Sidi Mohammed Ben Abdellah University - Faculty of Medecinee, FEZ, MAR; 3 Otorhinolaryngology, University Hospital Hassan II, FES, MAR; 4 ENT, University Hospital Center Hassan II, FES, MAR; 5 ENT, University Hospital, FEZ, MAR

**Keywords:** chronic otitis media, tympanic membrane perforation, surgery, cartilage, myringoplasty

## Abstract

Introduction: Myringoplasty remains a topical subject. Our study aims at analyzing the anatomical and functional results of cartilaginous myringoplasty, and also determining the main factors that could influence its results.

Materials and methods: A retrospective study of 51 cases of tympanic perforations operated at the ENT department of the Hassan II University Hospital of Fez between January 2018 and November 2021. Only the patients with exclusive cartilage myringoplasty were included. The anatomical and functional results of cartilage myringoplasty were evaluated and analyzed according to several variables. The statistical analysis was performed using SPSS Statistics software.

Results: The average age of our patients was 35, with a sex ratio of 2.45. The perforation was anterior in 58%, posterior in 12%, and central in 30% of the cases. The average pre-operative audiometric air bone gap (ABG) was 29.3 dB. The most commonly used graft was the conchal cartilage in 89% of cases. A complete cicatrization has been noticed in 92%, and at six months after surgery, a complete closure of the ABG has been observed in 43% of cases, a significant hearing improvement with an ABG between 11 and 20 dB in 24%, a hearing recovery with an ABG between 21 and 30 dB in 21%, and an ABG > 30 dB in 12% of the cases. A statically significant relationship (p<0.05) has been found between the functional or anatomical failure of the myringoplasty; the different predictive factors were: the young age (less than 16 years), the inflammatory state of the tympanic cavity, the anterior location, and large size of the perforation.

Conclusion: Cartilaginous myringoplasty provides good anatomical and auditory results. The pre-operative predictive factors, such as age, complete and sufficient drying of the ear, the size and location of the perforation, and the size of the used cartilage, should all be considered for a better anatomical and functional outcome.

## Introduction

Myringoplasty is a common practice in ENT due to the high incidence of infectious otologic pathology in the population. Myringoplasty aims to attain a dry and self-cleaning ear while preserving or even improving hearing [1]. During the past two decades, a certain number of matters have been raised and resolved, at least in part, that concern the donor material required for the graft, although the position of the graft concerning the manubrium of malleus is yet to be discussed. Our study is the first study of its kind in our area Fes-Meknes (Centre of Morocco) addressing cartilaginous myringoplasty; his aim is to analyze the anatomical and functional results of cartilaginous myringoplasty and determine the main factors affecting these results.

## Materials and methods

It is a retrospective study of 51 cases of tympanic perforations, operated at the ENT (Ear, Nose, and Throat) department of the University Hospital Center Hassan II of Fez (centre of Morocco), from January 2018 until November 2021. All the cases had benefited from a cartilaginous myringoplasty, performed by different otology surgeons. The study has been approved by the ethical committee of the Faculty of Medicine and Pharmacy of Fez and included only the patients operated for a sequelar tympanic perforation due to simple chronic otitis media. In addition, excluded from the study were all the incomplete files, the patients lost to follow-up, as well as: cholesteatomatous chronic otitis media with ossicular chain discontinuity, and the patients who have benefited from a functional surgery. We collected the demographic, clinical, and operative data retrospectively. The tone audiometry was analyzed before acquiring the pre-operative information randomly. Then, the second step consisted of an analysis of the surgery reports of each patient enrolled in the study arbitrarily in comparison to the data of the audiometry. The third step focused on the control of the operated patient after nine months to check: 

- The anatomical results: by assessing the integrity of the neo-eardrum, the absence or not of the refilling of the anterior angle, and the absence or presence of a tympanic medialization or lateralization.

- The functional outcomes: by Rinne’s test of the frequencies 0.5; 1; 2; 4 kHz in a post-operative audiometric test performed at six months after surgery at least, and the comparison with pre-operative Rinne’s test; the increase of air conduction is then calculated. The collected data were put on an Excel table (Microsoft Office 2013), the statistical analysis was performed using SPSS Statistics (v.22 for Windows), and the comparisons of percentages were done by Fisher’s exact test. Plus, we used Student's t test to compare the mean values and the various scores before and after surgery. A logistic regression was applied to examine the relationship between the success/failure of the graft and the variables; the significative values were p<0.05. 

## Results

The average age of our patients was 35, with extremes going from 16 to 54 years. There were 29% of men and 71% of women. The sex ratio was 2.45. The periods of consultations were variable, ranging from six months to several years. We found in the patients’ history: repetitive otitis in 10% of the cases, trauma in 8%, and allergic rhinitis in 13%. The main functional symptoms were: purulent otorrhea in 44 cases (which represents 87.5%), hearing loss in 40 cases (80%), tinnitus in 19 cases (37.5%), and otalgia in 37 cases (72.5%). 

The otoscopic examination highlighted: a bilateral perforation in 30% of the cases and a unilateral perforation in 70%; the left side was the most affected one, which represents 64% of the cases. The perforation was anterior in 58%, posterior in 12%, and central in 30% of the cases; the perforation was important in 73% and small in 27% of the patients. The bottom of the tympanic cavity was dry in 74.5%, inflammatory in the rest (notably: humid in 15.5% of cases, and polypoid in 7.8%), and infected in 2.2%. Acoumetry highlighted conductive deafness for 95% of cases, and it was subnormal for 5%. The rhinoscopy allowed the discovery of inflammatory rhinitis for 13% of cases, as well as a septal deviation for three patients.

The pure-tone audiometry has shown: a conductive deafness for all cases, with an average loss of less than 30 dB for 75% of the cases, and more than 30 dB for 25%. The average audiometric air bone gap (ABG) before surgery was about 29.3% with extremes from 6 to 45 dB. The surgical approach was retro auricular in 92% of cases and endaural in 8%. The most used graft was the conchal cartilage for 89% of the patients, while for the other 12%, it was the tragal cartilage.

The anatomical control of our patients has been performed using otoscopy after an average period of 13.5 months (3-24 months). Complete wound healing was noticed for 92% of the patients, while the other 8% witnessed a residual perforation. At about six months after surgery, and out of any infectious context, we have noticed a complete closure of the air-bone gap at 43% of cases, a notable hearing improvement with a ABG between 11 and 20 dB at 24%, an auditory recovery with a ABG between 21 and 30 dB at 21%, and an ABG>30 dB for 12% of the cases.

To determine the different predictive factors of both success and failure of cartilaginous myringoplasty, we used different criteria (as shown in Table 1): age, gender, medical history, the state of the middle ear, the location and size of the perforation, the perforated side, the drying-up period, and the nature of the chosen cartilage. The relationship was statistically significant (p<0.05) between the failure of the cartilaginous myringoplasty and its predictive factors: early age (less than 16 years old), the inflammatory condition of the middle ear, the anterior siege, and the important size of the perforation.

**Table 1:** Prognostic factors of the anatomical and functional results.

	Assessment criteria	Anatomical failure		Auditory failure	
		%	p	%	p
Age	Younger than 16 years	4.3	0.007	33	0.23
	Older than 16 years	5		10	
Gender	M	8.3	0.52	31.5	0.71
	F	16		38.8	
Medical history	Yes	5.3	2.1	18.1	0.45
	No	3.2		25	
State of the tympanic cavity	Dry	5	0.03	41.6	0.66
	Inflammatory	8		32	
Location of the perforation	Anterior	5.7	1.7	4.7	0.02
	Others	4		4.5	
Perforation side	Right	17	0.34	28	0.4
	left	20		50	
Perforation size	Small	6	0.018	35	1.8
	Big	5.4		31	
Drying period	Less than 3 months	23.1	0.32	60	0.44
	More than 3 months	8.3		34.3	
Cartilage	Tragal	3	0.2	2.4	0.38
	Conchal	23		6.2	

## Discussion

The study of Darouassi et al. [2] gathered 140 patients over six years, with an average age of 34, and Shoman’s study [3] focused on 250 patients over 4 years, with 46 years as an average age. However, the mean age of the patients -- in our series -- ranges from 16 to 54, with an average of 35 years. The gender distribution was variable in the literature, even though in the majority of the series, there was a female predominance [2-5]. Altuna et al. [6] revealed some pathological backgrounds in 99 cases: 52 of them had already undergone a former surgery (49 cases of myringoplasty and three cases of grommet insertion). The affected side in our study was ‘left’ for 64% of our patients, which corresponds to the majority of studies (40.72). Darouassi et al. [2] and Zhang et al. [4] reported that the subtotal perforation was the most frequent representing -- respectively -- 47% and 32.8%. Shoman [3] and Karabulut et al. [5] highlighted that the anterior perforation was the most common, being respectively 40% and 35.7%, which goes in line with our study. 

The size of the perforation has been largely dealt with in the literature; Shoman [3] reported in his series that the perforation was small in 12% of the cases, average in 41%, and big in 47%. Plus, Zhang et al. [4] reported in his series that the perforation was small in 15.6% of the cases, average in 47.9%, and big in 36%. Usually, the surgery occurs when the mucosa of the tympanic cavity is dry. In the series of Darouassi et al. [2], the mucosa was dry in 100% of the cases. Zhang et al. [4] highlighted a dry and normal mucosa in 421 cases and humid in 102. In our study, the used graft was the conchal cartilage for 88% of cases, and the tragal cartilage for the other 12%. The majority of the literature’s cases reported that the most used cartilage is the tragus for 100% of the patients [3-5]. The rate of anatomical success of our patients was 92%, which is comparable to the rates cited in the literature for cartilaginous myringoplasty: 93% in the study of Yurttas et al. [7], 92.3% in the study of Onal et al. [8], and 90.7% in Elboukhari [9]. In addition, age is often cited as a key prognostic factor in the assessment of tympanoplasty for children [10]. In recent meta-analyses, adults represent a higher success rate of myringoplasty than children. Within the pediatric population itself, these results vary between bigger and small children [11], which corresponds to the results of our study: there is a significant difference in the success rate between the children under 16 (95.7%), and the teens and adults over 16 (95%) (p<0.01).

The clear otorrhea that accompanies the sequalae tympanic perforations of otitis media, can affect the success rate of myringoplasty. Gersdorff et al. and Pignataro et al. obtained better results when operating dry ears, and then the two recommended a medical treatment of draining ears to monitor the inflammatory process before myringoplasty [12-13]. Interesting fact, Pignataro et al. have concluded that a dry ear is the most significant factor for the success of the myringoplasty [13]. In our study, the success rates depending on the size were statistically significant (value of p is 0.018). In literature, opinions differ on this viewpoint. Lee et al. [14], in their study, entitled: ‘Myringoplasty: does the size of the perforation matter?’, have shown that the success rate in small perforations was 74%, while in bigger perforations, that rate was only 56%. In the study of Das et al. [15], they found a 100% success rate in small perforations and 42.9% in bigger ones; they also considered the size of the perforation a significant factor influencing the success of myringoplasty.

Anterior perforations are technically difficult to proceed with, and vascularization is usually poor. Many authors have mentioned that the site of the perforation could have a greater influence on the prognosis of surgery: Bhat and De [16] and Gersdorff et al. [12] noted in their study that the success relies on the site of the perforation. In our study, we noticed that the success rate of central and posterior perforations was better than in anterior ones, but it was not statistically significant.

The drying period of the ear before myringoplasty was one of the several factors studied by Onal et al. [8]. To determine their impact on the surgery’s outcomes, they noted that a myringoplasty was more likely to succeed if the ear had been dry for a longer period; they have observed that every time it has been dry for less than one month before surgery, the success rate was 60%, and if it had been dry for more than one month, that rate goes up to 82%, and the difference was statistically not significant but close to significance level (p=0.067). On the other hand, a few studies have demonstrated the positive impact of ear draining on the grafting’s success. Mills et al. reported a success rate -- after myringoplasty -- of 82% and 83% in humid and dry ears, respectively [17].

The type of the surgical approach did not impact des results of myringoplasty in our study (93% of success for the retro-auricular approach, and 91.5% for the endaural one), which corresponds to the study of Elboukhari [9] who has demonstrated a 91.4% success rate of the retro-auricular approach vs. 90.5% for the endaural.

There is no mutual agreement on the selection of the grafting materials for myringoplasty; the selection depends completely on the surgeon’s experience and preference. Over the years, many materials (natural and synthetic) have been tested, but very few studies dealt with the results [10].

The cartilage provides a good rate for the tympanic membrane sealing (90.80%), compared to the temporal fascia (88.00%) [5]. Tek et al. [18] found out that cartilage grafting was significantly better than temporal aponeurosis. 

The tragal cartilage is a perfect material for the reconstruction of the tympanic membranes due to its thinness, its flat shape, and the sufficient quantity to cover the whole tympanic membrane [19]. The only advantage of fascia -- compared to the cartilage -- might be the ability to detect iatrogenic cholesteatoma [19].

In our study, the mean air conduction gain -- after one year -- shifted from 34.5 dB before surgery, to 19.2 dB after, and an improvement of the postoperative residual ABG from 27.5 dB before surgery to 8.9 dB later, which corresponds to the results of the literature (Table 2). In the study of Shoman [3], the post-operative residual ABG has significantly improved, from 29.2 dB before surgery to 9.6 dB after. Moreover, the study of Alain et al. [20] demonstrated that the mean air conduction gain changed from 29.7 dB before surgery to 9.1 dB after surgery. Similarly, the study of Zhang et al. [4] showed that the mean air conduction gain also moved from 38.4 dB before surgery to 25.1 after, and the residual postoperative air-bone gap had also improved from 22.4 dB before to 9.1 dB after.

**Table 2:** Comparison of functional results. ABG, air borne gap

		Alian et al. [21]	Shoman [3]	Zhang et al. [4]	Our study
The mean air conduction gain	Pre-operative	37,7 dB	-	38.4 dB	34.5 dB
	Post-operative	10.6 dB	-	25.1 dB	19.2 dB
The mean residual ABG	Pre-operative	29,7 dB	29,2 dB	22.4 dB	27.5 dB
	Post-operative	11.3 dB	9,6 dB	9.1 dB	8.9 dB

When it comes to the factors affecting the functional results, Salviz et al. [21] noticed a statistically significant difference concerning the air conduction gain, whether their age is more or less than 16 (9 dB vs. 13 dB, p = 0.022). Similarly, Vartiainen [22] showed that the young age is considered a functional failure factor for myringoplasties (the post-operative average AGB is less than 20 dB in 84% for adults and only 41% for children). However, several authors have not discovered the impact of age on the audiometric results of myringoplasty [3], and this corresponds well with the results of our study: age does not affect functional outcomes (p > 0.05). Technically speaking, the perforations of the anterior siege are difficult to access and to put the graft in. However, recent studies have not mentioned poorer results with anterior perforations. Pignataro et al. have noticed that the perforation site has no impact on both anatomical or auditory results [13]. Yet in our study, there was a significant difference between the functional results of patients with anterior perforations and the ones with other locations (p=0.02).

When it comes to the size of the tympanic perforation, the literature’s data are also contradictory; some studies showed that subtotal perforations have poor prognosis than the smaller ones, yet this was not confirmed by many recent series [23]. Concerning the drying period of the ear, several authors recommend a three-month period [12]. Regarding the nature of the used graft, the cartilaginous graft represents -- for Roger et al. -- the material of choice in circumstances where grafting is problematic (such as perforations that are large or tend to be inflammatory, and/or dysfunction of the eustachian tube) due to its rigidity and resistance to the necrosis [23]. It is true that our study agrees in general with the data of the literature, but it remains a retrospective study with a limited number of patients.

## Conclusions

Cartilaginous myringoplasty provides good results on both anatomical and auditory levels. Age, a sufficient and complete drying of the ear, the size and location of the perforation, and the size of the used cartilage, must all be taken into consideration as preoperative predictive factors to achieve a complete and durable sealing of the tympanic membrane, as well as satisfying auditory outcomes after a cartilaginous myringoplasty. The next goal is to confirm these results on a larger scale, and in the long term, to develop this technique in ambulatory settings, or even a suitable consultation setting.
